# P-1164. Prevalence of invasive Candidiasis and antifungal resistance in neonates: a multicenter three-year retrospective analysis from two large Mexican NICUs

**DOI:** 10.1093/ofid/ofae631.1350

**Published:** 2025-01-29

**Authors:** Abraham P Garza-Castro, Ana Ballesteros-Suarez, Lindsay A Concha-Mora, Ericka Paulina Prieto-Baca, Oscar Tamez-Rivera

**Affiliations:** Residencia de Pediatría, Programa Multicéntrico de Especialidades Médicas. ITESM-SSNL. Tecnológico de Monterrey, Escuela de Medicina y Ciencias de Salud., Santiago, Nuevo Leon, Mexico; Departamento de Medicina, Tecnológico de Monterrey, Escuela de Medicina y Ciencias de la Salud, Monterrey, México, Monterrey, Nuevo Leon, Mexico; Residencia de Pediatría, Programa Multicéntrico de Especialidades Médicas. ITESM-SSNL. Tecnológico de Monterrey, Escuela de Medicina y Ciencias de Salud., Santiago, Nuevo Leon, Mexico; Departamento de Medicina, Tecnológico de Monterrey, Escuela de Medicina y Ciencias de la Salud, Monterrey, México, Monterrey, Nuevo Leon, Mexico; Tecnologico de Monterrey, Escuela de Medicina y Ciencias de la Salud, Monterrey, Nuevo Leon, Mexico

## Abstract

**Background:**

Candida infections are increasingly recognized as clinically relevant pathogens in NICUs, particularly among preterm and low birth-weight infants (LBWI). Due to the emergence of Fluconazole-resistant *Candida* spp, current treatment guidelines of neonatal invasive Candidiasis (IC) suggest Amphotericin B as first line therapy, leaving Fluconazole as an alternative agent. Microbiological surveillance focused on fungal etiologies in Mexican NICUs is scarce, which may lead to inappropriate antifungal use.

**Figure:**
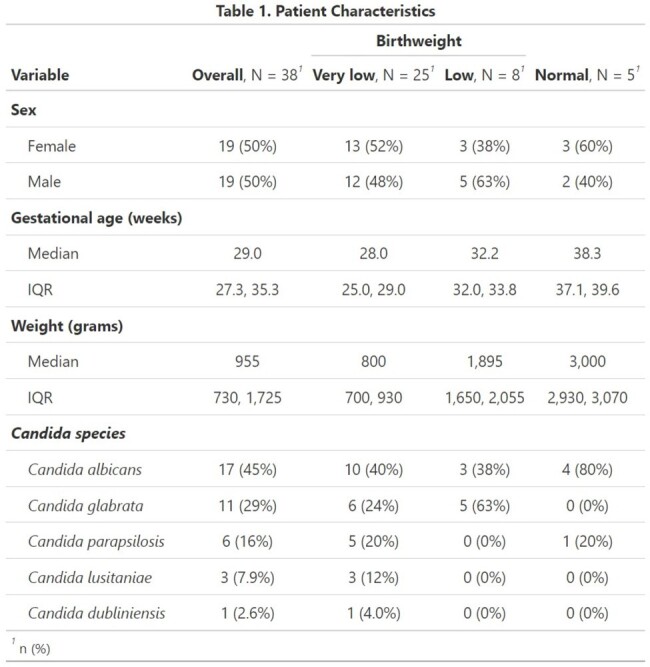

**Methods:**

Retrospective multicenter study conducted in the NICUs of the two pediatric reference hospitals in NL, Mexico. Clinical and microbiological data of all newborns ≤28 days diagnosed with blood culture-proven invasive Candidiasis from 2021-2023 were collected. LBWIs were defined as newborns < 2500 g at birth, and very LBWI (VLBWI) as < 1500 g. Descriptive statistical analyses were performed to determine the frequency of *Candida* spp and frequency of antifungal resistance.

**Figure:**
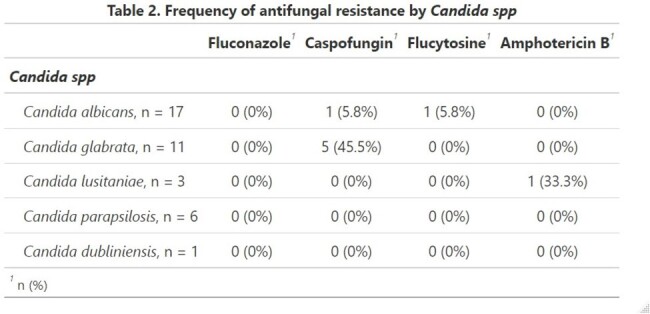

**Results:**

During the studied period, 928 positive blood cultures were included. Only 38 blood cultures (4%) were positive for *Candida* spp. No outbreaks occurred during the studied period. Twenty one patients (75%) were preterm. Mean gestational age was 29 weeks. Most patients (55%) were VLBWI (Table 1). All episodes were classified as late-onset sepsis. The most common isolated species were *C. albicans* (45%), followed by *C. glabrata* (29%), and *C. parapsilosis* (16%). No Fluconazole resistance was observed (Table 2). Caspofungin resistance was found in 5 *C. glabrata* isolates and 1 *C. albicans*. No cases of endophthalmitis or endocarditis were documented.

**Conclusion:**

We report a low prevalence of blood culture-proven neonatal IC (4%) in two large Mexican NICUs during the studied period. Most patients were LBWI. This is consistent with international data. C. albicans was the most common isolated species. Based on the local epidemiology in two large NICUs of NL Mx, Fluconazole may be considered as a first line antifungal agent instead of Amphotericin B. Local microbial epidemiology and antifungal susceptibility patterns should be considered for the empirical treatment.

**Disclosures:**

**All Authors**: No reported disclosures

